# Maternal and neonatal health impact of obstetrical risk insurance scheme in Mauritania: a quasi experimental before-and-after study

**DOI:** 10.1093/heapol/czw142

**Published:** 2016-10-20

**Authors:** Aline Philibert, Marion Ravit, Valéry Ridde, Inès Dossa, Emmanuel Bonnet, Florent Bedecarrats, Alexandre Dumont

**Affiliations:** 1 Interdisciplinary Research Centre on Well-being, Health, Society and Environment (Cinbiose), University of Quebec in Montreal, Montreal, Québec, Canada; 2 Research Institute for Development, Université Paris Descartes, COMUE Sorbonnes Paris Cité, UMR MERIT, Paris, France; 3 IRD, CEPED, UMR 196, Université Paris Descartes—Institut de Recherche pour le Développement (IRD), Paris, France; 4 School of Public Health (ESPUM), University of Montreal, Montreal, Quebec, Canada; 5 University of Montreal Public Health Research Institute (IRSPUM), Montreal, Quebec, Canada; 6 UMR IDEES CNRS 6266, Université de Normandie/IRD RESILIENCE 236, Caen, France; 7 Agence Française de Développement (AFD), Evaluation Unit, Research and Knowledge Developpement, Paris, France

**Keywords:** Community-based health insurance, infant mortality, maternal health, Mauritania, Sub-Saharan Africa

## Abstract

A variety of health financing schemes shaped on pre-payment scheme have been implemented across Sub-Saharan Africa (SSA) to address the Millennium Development Goals (MDGs). In Mauritania, the Obstetric Risk Insurance package (ORI) focusing on maternal and perinatal health has been progressively implemented at the health district level since 2002. Here, our main objective was to assess the effectiveness of the ORI in increasing facility-based delivery rates, as well as increases in family planning, antenatal and postnatal care, caesarean delivery and neonatal health, from demographic and health survey data between 2002 and 2011. We also examined whether the effects of the ORI varied between strata of the population. The study was based on a quasi-experimental before-and-after design to assess the causal link between availability of ORI and increase in use of maternal health services and neonatal mortality. In combination with geographical information system, difference-in-differences and odd ratio approaches were used to address our objectives. Indicators of access to care for pregnant women and neonatal health and improved in both non-intervention and intervention groups during the study period. There was no global effect of the availability of ORI on facility-based delivery rates, nor on the use of antenatal and postnatal care services, except for qualified antenatal services. However, delivery rates in local health centres with ORI increased more rapidly than in those with no ORI, the contrary was shown for hospitals. Caesarean delivery and family planning decreased with ORI. Although late neonatal mortality rates remained low in the country, a significant decrease was seen in districts without ORI. Except for some strata of the population, ORI has not really met its objective of attracting more pregnant women towards facility-based health care.

Key Messages:

- Indicators of maternal and neonatal health and access to care substantially improved in all health districts independently of the availability of the ORI.
- Although there was no global effect of the availability of ORI on facility-based delivery rates, nor on the use of antenatal and postnatal care services, there was significant evidence of increasing access to birth deliveries in nearby health centres at the expense of hospitals in the districts providing ORI.
- Regardless of the availability of ORI, the education level of women strongly influences the use of maternal health services.
- Neonatal mortality rates remained low and no global decrease was recorded with the availability of ORI.

## Introduction

Arose out of the United Nations Declaration of 2000, the Millennium Development Goals (MDGs) have stimulated interest and encouraged funding for programs that aimed at reducing child mortality (MDG-4) and improving maternal health (MDG-5) (Waage *et al.* 2010; Bryce *et al.* 2013). Among the different programs introduced, some health financial strategies have been initiated in Sub-Saharan Africa (SSA) to increase access to quality and responsive maternal and perinatal health care, while providing financial protection (De Allegri and Sauerborn 2007; Ridde *et al.* 2011; Smith and Sulzbach 2008). Indeed, large amounts and unpredictability of costs related to maternity remains one of the major barriers for seeking health care (Ensor and Ronoh 2005; Kurfi *et al.* 2013) as they absorb a significant amount of the financial capital in the household, sometimes generating financial catastrophic expenditure (Arsenault *et al.* 2013).

Among a variety of health financing systems, many pre-payment scheme designs have been introduced at local and/or national level across SSA (Carrin *et al.* 2005; Ensor and Ronoh 2005; Smith and Sulzbach 2008). Basically, a pre-payment scheme aims at increasing health care utilization but also at providing financial risk protection (Smith and Sulzbach 2008). The main difference with an insurance scheme lies in risk sharing, which is necessary in this case but not in the case of a pre-payment scheme. They are non-profit systems, based on voluntary affiliation, collective pooling of resource and risk-sharing, and flat membership premiums (Carrin *et al.* 2005; Onwujekwe *et al.* 2009, 2010). The pre-payment scheme has been progressively incorporated into the national health financing strategies of Benin, Ghana, Rwanda, Senegal and Tanzania with relative success (Smith and Sulzbach 2008; Borghi *et al.* 2015).

Following the implementation of pre-payment schemes in SSA, there has been evidence of increased utilization of pregnancy-related care, increased facility-based deliveries and improved financial protection due to reduced out-of-pocket expenditure in Guinea, Mali, Mauritania, Nigeria, Rwanda and Senegal (Adinma, Nwakoby and Adinma 2010; Chankova, Sulzbach and Diop 2008; Ndiaye *et al.* 2008; Schneider and Diop 2001; Smith and Sulzbach 2008). In contrast, there are a few studies that failed to identify an effect of pre-payment schemes. For example, no significant difference in overall antenatal care services, facility-based deliveries and/or maternal mortality were reported among members and non-members of the Nouna health district in Burkina Faso (Hounton *et al.* 2012). While both Ghana and Rwanda implemented national social health insurance (NHIS) respectively in 2003 and 2005 and are both characterized by very good progress towards universal health coverage, the effects on maternal seeking behaviour have been partially achieved (Brugiavini and Pace 2010; Mensah *et al.* 2010; Singh *et al.* 2011; Blanchet *et al.* 2012; Frimpong *et al.* 2014; Ahiadeke 2015). Although >90% of pregnant women were covered in the two countries, only 67% had facility-based deliveries in Rwanda and 57% in Ghana (Speizer *et al.* 2014; Twahirwa 2008; Lu *et al.* 2012; Abrokwah *et al.* 2014). Finally, some results on increased antenatal care seeking and reduced out-of-pocket expenditure were inconclusive given differing findings between studies (Brugiavini and Pace 2010; Chankova *et al.* 2008; Mensah *et al.* 2010; Singh *et al.* 2011; Smith and Sulzbach 2008).

In Mauritania, Obstetric Risk Insurance (ORI) progressively implemented at the health district level between 2002 and 2011 was based on pre-payment scheme principles and focused on increasing quality and access to both maternal and perinatal health care. Our paper aims to assess the effectiveness of the ORI in increasing the use of facility-based delivery care, as well as family planning, antenatal and postnatal care, caesarean delivery, and neonatal health. We also examine whether the effects of ORI vary according to household wealth and women’s level of education to understand the equity implications of the ORI approach.

## Methods

### Public health system in Mauritania

Located in Western Africa, Mauritania has a human development index ranking of 156 out of 186 countries in 2014 (source on UNDP website). In 2013, the health expenditure represented 3.8% of the GDP according to the World Bank, averaging in a health expenditure per capita of 48.96 US$. The Maternal Mortality Ratio (MMR) in Mauritania was 602 per 100 000 live births in 2015 (based on World Bank data). In 2015, neonatal mortality was 36‰ and under-five mortality of 85 ‰ (World Bank data).

Mauritania's public health system is organized in a common hierarchical pyramid: the central level, represented by the Ministry of Health; the intermediate level, made up of Regional Health Directorates («Directions Régionales de l’Action Sanitaire»: DRAS) situated in the 13 Wilayas or regions; and the peripheral level, composed of 53 health districts re-grouping health centres and health posts (Renaudin *et al.* 2007, 2008). In 2O13, there were 53 health districts distributed in 13 regions.

The public health care facilities are divided into three levels. At the first level, health posts provide primary health care and are generally run by nurses. These include antenatal visits, normal vaginal delivery, basic neonatal care, first-line treatment of obstetric complications (parenteral administration of oxytocin, anticonvulsants and antibiotics), monitoring of children under 5 years and family planning. At the second level of care, health centres provide essential services cited above and some laboratory tests. Some health centres have a general surgical unit and radiology. At the third level, regional or national hospitals provide comprehensive emergency obstetric care (EmOC), including transfusions and caesarean sections.

### Health districts and participants

The present study examined health service utilization and neonatal mortality, using nationally representative data from four recurring community surveys (ICSs) carried out in Mauritania: the Demographic and Health Survey (DHS) in 2001 (MEASURE DHS); the National Survey on Infant Mortality and Malaria (NSIMM) in 2003; and the Multiple Indicator Cluster Surveys (MICS) in 2007 and 2011. In all those four surveys a two-stage cluster random sampling was carried out. The selection was conducted among census districts at the first stage and among households at the second stage. According to sampling methods used in the surveys, households were representative of the whole population in Mauritania. We utilized data on all interviewed women aged 15–49 of each selected household to describe levels of contraception use. To study neonatal outcomes and levels of healthcare services use during pregnancy, delivery and the postpartum, only women who delivered a live-born child in the last two years before the date of the interview were retained for analysis. The time period considered for the present study extended from July 2000 to November 2011.

Complementary information for mapping (geographical information system or GIS) was provided by the Spanish cooperation «La Agencia Española de Cooperación Internacional para el Desarrollo» or AECID, and completed by available data from the Open Street Map platform (OCHA HDX website). The QGis software was used for mapping spatial distribution by health district.

### Study design

The present study was based on a quasi-experimental adjusted before-and-after study design (Grimshaw *et al.* 2000). The unit of intervention was the health district and the unit of analysis was the women or neonates. We compared the levels of health service utilization and neonatal outcomes between women who lived in a district participating to the ORI and those who lived in a district with no ORI. The date of the first implementation of the ORI in the district of residence was used for ‘before’ and ‘after’ analyses during the study period (2000–2011). This implementation date varied from 2002 to 2011 among districts that participated to the ORI. Among the 15 participating districts and the 48 eligible health care facilities, 44 (92%) received ORI between 2008 and 2011. For the purpose of comparative analysis, 1 January, 2008 was used as an arbitrary date for ‘before’ and ‘after’ analysis in districts with no ORI (non-intervention group) because that date corresponds to the medium of the time period and that the majority of participating health care facilities started the ORI package after 2008 in the intervention group. It should be mentioned that the available databases did not indicate whether the respondents subscribed to the ORI scheme. Accessible information for ORI implementation was limited to the district level. For analysis purposes, we assumed that a woman who lived in a district where the ORI was available had better access and higher probability to join the ORI scheme.

Data used from the ICSs were collected in districts with or without ORI contemporaneously using consistent methods before and after the implementation was introduced. The National Office of Statistics (NOS) monitored all information collected in those four databases. Common used data merging procedures were carried out to adjust for differences and inconsistencies among data definition, format and methods in order to make the data mutually compatible. Harmonization of data from the four household surveys allowed us to consolidate information on maternal and neonatal health for 10414 births between 2000 and 2011. Data collected in the 48 health care facilities were integrated into one database after merging the four community surveys cited above. The merging of data was done through simple recoding of data categories, aggregation of data or unit transformation. The information included use of health services during pregnancy, labour and postpartum, as well as main characteristics of households, women and neonates (Table 1).

**Table 1:** Distribution of outcomes in the four community-based surveys

	DHS 2001	NSIMM 2003	MICS 2007	MICS 2011
Mean size of households	7.78	–	7.17	7.38
No of respondent women	7728	5211	12549	12754
Mean age of women	27.88	28.02	28.44	28.59
No of women who delivered a live-born child in the last two years before interview	1979	1267	3539	3629
Modern methods of contraception	261 (3%)	–	588 (5%)	756 (6%)
Antennal care: at least one visit	1378 (33%)	1262 (99%)	2545 (72%)	3070 (84%)
Antenatal care by qualified staff	657 (33)	917 (72%)	2545 (72%)	3011 (83%)
Antenatal care: at least four visits	347 (18%)	–	–	1895 (52%)
Facility-based delivery	1061 (54%)	–	1942 (55%)	2253 (62%)
Caesarean delivery	68 (3%)	–	–	306 (8%)
Postnatal care: at least one visit	291 (15%)	–	468 (13%)	1226 (34%)
Early neonatal mortality (up to 7 days)	48 (2%)	37 (3%)	–	59 (2%)
Late neonatal mortality (up to 28 days)	56 (3%)	45 (4%)	–	71 (2%)

Abbreviations: DHS: Demographic and Health Survey; NSIMM: National Survey on Infant Mortality and Malaria; MICS: Multiple Indicator Cluster Surveys . NSIMM in 2003 did not provide data to estimate the use of modern contraceptives, the number of antenatal visits, the place and mode of delivery and the use of postnatal care. MICS 2007 did not provide data to estimate the number of antenatal visits and neonatal outcomes.

Data merging was done by using SPSS statistical software (version 20.0; SPSS, Inc, Chicago, IL)

### Intervention

The ORI, which was implemented at the health district level, targeted pregnant women attending antenatal care in ORI-participating health care facility. The scheme is based on a flat fee pre-payment. Any woman attending antenatal care is given the choice to enrol on a voluntary basis. If she agrees, she pays a fixed premium and automatically becomes member for all maternity-related care in any participating health care facility of the district where she was enrolled. The premium can be paid in one or two instalments and varies from 5500 ouguiyas (14 euros) in districts outside of the capital Nouakchott to 6500 ouguiyas (16 euros/18 USD) in Nouakchott. This remained lower than in other public maternities where a delivery cost varies between 11 and 30 euros/12 and 34 USD for a caesarean-section up to 200 euros/224 USD. (Renaudin, Prual *et al.* 2007; Vinard 2011).

Maternity-related care for the ORI membership included at least: four antenatal visits; all prophylactic treatments; one blood test (haemoglobin level, blood group and rhesus); one urine test (proteinuria, glycosuria) at each antenatal visit; one ultrasound scan during the first trimester; treatment for any pathologies related to pregnancy and delivery, skilled delivery and emergency obstetrical care (EmoC) if needed, including caesarean section; ambulance transportation to a higher level of health care; hospital care if transferred; and one postnatal visit (Renaudin *et al.* 2007, 2008).

The ORI was firstly implemented in 2002 in two health districts of Nouakchott (Sebkha and El Mina) and since then, has extended still further in other districts outside of the capital. At the end of 2014, the ORI was available in 144 out of 627 public health care facilities (93/528 posts, 40/81 health centres and 11/18 hospitals). The selection of participating health care facilities was based on a series of conditions, such as estimation of the needs in the area and rate attendance at the facility in the targeted districts (Renaudin *et al.* 2007, 2008). The implementation of the ORI was supposed to be accompanied by an important step in improving the quality of care and in raising the awareness of the population of the district before the ‘opening’, a technical support regarding supervision of medical staff and by the existence of a health insurance manager. In parallel, drugs, supplies and essential equipment for appropriate obstetric care are provided to participating health care facilities before starting implementation (Renaudin *et al.* 2007). The benefits generated by the enrolment fees help to operate the health care facilities. Those include essential drugs and supplies and incentives paid to health workers (Renaudin *et al.* 2007, 2008); the fixed salary of health workers being ensured by the government and new equipment by the government or donors. In general the ORI starts in concomitance in the health facility with the highest level of care facility (e.g. the referral hospital), and some with lower level of care (e.g. heath post or center) in the participating districts.

### Outcomes

The primary outcome was the facility-based delivery rate. All deliveries occurring in a health care facility were considered as facility-based. Public health care facilities include hospitals, district health center and health post.

Secondary outcomes included population-based rates of current use of modern contraceptives, antenatal and postnatal care attendance, caesarean delivery and neonatal mortality. We considered three indicators for antenatal care attendance: (i) at least one visit, (ii) at least one visit with trained medical staff and (iii) four visits or more during pregnancy. Two time periods were considered for neonatal mortality: early (0–6 days of age) and late neonatal mortality (7–27 days of age).

### Statistical analyses

The sample size was calculated in such a way to maximize statistical power, while demonstrating a significant difference in facility-based delivery rate of 30% between the non-intervention and intervention group (expecting rate of 80% - average rate of 50% before the ORI was introduced). Based on an average of 145 women per district who delivered in the past two years before the date of the interview, an observed intra-cluster correlation coefficient (ρ) of 0.30, and an alpha error of 0.05, the minimum number of women required per group to reach 80% statistical power was 1245 (Software ACluster-design®2005, version 2.0, World Health Organization).

We assessed the effect of the availability of the ORI in the district on facility-based delivery rate using multivariate generalized estimating-equations extension of logistic regression models to account for the clustering of women within districts. Changes in the odds of facility-based delivery in the two groups between ‘before’ period and ‘after’ period were compared with the use of an adjusted odds ratio (with 95% confidence interval) for the interaction between group (district with ORI vs district with no ORI) and time period (before vs after). The difference in rate changes between the two groups and the adjusted odds ratios for interaction measured the intervention effect with the difference-in-difference approach, which was adapted to multivariate hierarchical analyses of clustered binary outcomes (Card and Krueger 1994). Two-tailed *P* values of less than 0.05 were considered to indicate statistical significance.

All primary analyses include adjustments for pre-specified factors that could modify the effect of the intervention. These included adjustments for district localization (Nouakchott vs other region), level of the ORI adhesion (ratio of the adhesion to the expected birth deliveries) in the targeted district (<50, 50–79 and 80% and more), rural versus urban context, highest level of health facility in the district of woman’s residence (post, centre or hospital), highest level of health facility in the municipality of woman’s residence (post, centre or hospital), household size, household wealth category (quintiles), woman education level (none, Koranic education, primary, secondary level and more), marital status (married vs non married), maternal age categories (<18, 18–35 et 35+ years), parity and multiple pregnancy (Y/N). Household economic status or wealth index was estimated on the value of the selected household’s asset ownership, such as commodities purchased in the markets (household assets, e.g. means of transportation, possession of a television, phone), household size and housing characteristics. The wealth index of households was considered as a composite measure of living standards. We collected data on household ownership of selected assets and used principal components analyses to generate household asset-based proxy wealth indices, as commonly described in other studies (Vyas and Kumaranayake 2006; Howe *et al.* 2008; Gunnsteinsson *et al.* 2010). The wealth index was divided into quintiles for better discrimination (from the lowest Q1 to the highest quintile Q5).

To assess whether the intervention effect varied between subgroups of the population as household wealth and/or woman education level, we tested the corresponding three-way interactions: subgroup x intervention x time. Subgroup specific intervention effects were reported for outcomes with significant three-way interactions (tests were two-tailed, with *P* < 0.05 considered to indicate statistical significance).

Pre-specified secondary outcomes were analysed by means of methods similar to those used for the primary outcome. Analyses of contraceptive use were based on all women age 15–49; analyses of health service use during pregnancy, labour and post-partum were restricted to women who delivered a live-born child in the last two years before the date of the interview; analyses on neonatal mortality were restricted to live-born child.

All analyses were performed with the use of Stata 12 software (StataCorp. 2009. Stata Statistical Software: Release 11.0. College Station, TX: Stata Corporation).

## Results

**Figure 1:**
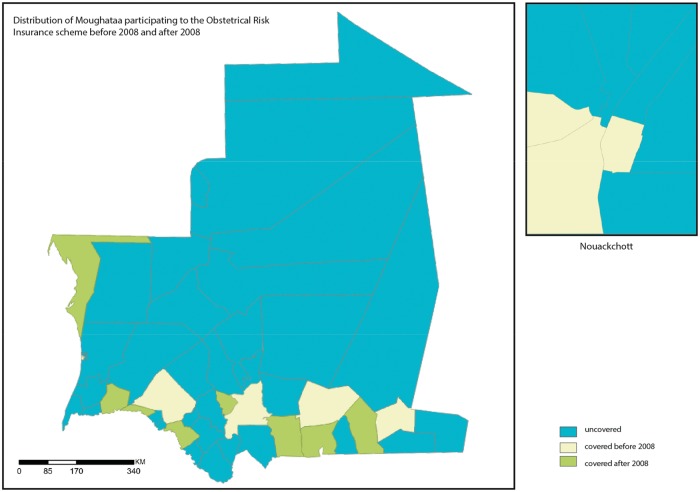
Distribution of the health districts that participated to the Obstetrical Risk Insurance (ORI) scheme before and after 2008.

**Figure 2:**
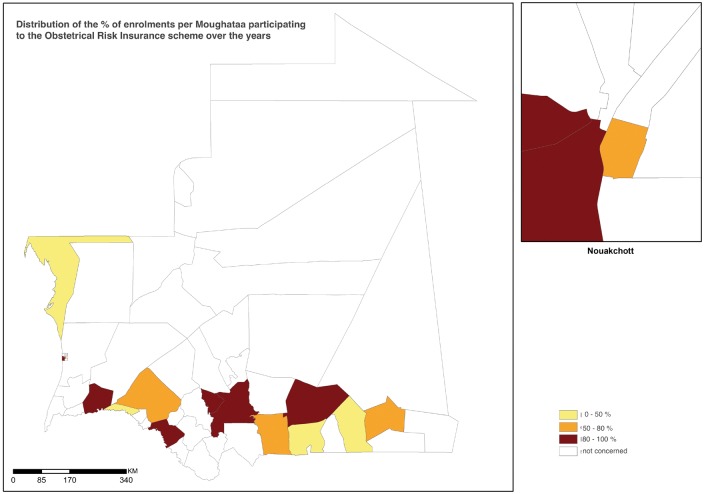
Distribution of the level of women enrolmnent per heath district. Note: The level of women enrolment per health district is estimated as the mean of annual percents of enrolments for the period between 2008 and 2010 for possible comparison. Annual percent of enrolments is the total number of women who paid the fixed premium during pregnancy divided by the expected number of pregnancies in the Moughataa.

Figure 1 illustrates the heterogeneous distribution of health districts participating to the ORI scheme before and after January 2008. The implementation of the ORI was restricted to Nouakchott and the southern part of the country. It started in Nouakchott (2002) and then was expanded to other districts in the southern regions from 2005. Table 2 shows the level of enrolments per district (mean proportion of pregnant women who paid a fixed premium between 2008 and 2010) and Figure 2 illustrates the distribution of the proportion of enrolment for the Districts that participated to the ORI. Among the 15 districts where the ORI was implemented, the mean adhesion rate in the district reached 81.5% but this rate was <80% *ranging from 35 to 70%) in seven districts.

**Table 2:** Distribution of the level of women enrolment per health district

Region	District	Date of the first implementation ^a^	Mean of annual percents of enrolments ^b^(%)	Level of Enrolment ^c^(%)
Assaba	Kiffa	2005	101.23	> =80
Assaba	Guerrou	2008	164.47	> =80
Brakna	Aleg	2007	60.74	50-80
Brakna	Boghe	2009	35.04	< =50
Dakhlet nouadhibou	Nouadhibou	2008	43.44	< =50
Gorgol	Kaedi	2008	84.41	> =80
Hodh el chargui	Nema	2005	76.58	50-80
Hodh el chargui	Timbedra	2009	36.04	< =50
Hodh el gharbi	Aïoun	2005	87.92	> =80
Hodh el gharbi	Kobonni	2010	40.35	< =50
Hodh el gharbi	Tintane	2010	56.94	50-80
Nouakchott	El mina	2002	94.30	> =80
Nouakchott	Sebkha	2002	230.57	> =80
Nouakchott	Arafat	2004	62.45	50-80
Nouakchott	Ryad	2010	99.31	> =80
Total			81.52	> =80

a Date of the first implementation in the district is the year when the first health care facility (hospital or health center) proposed to pregnant women to enrol the obstetric risk insurance (ORI) during her first antenatal visit.

b The mean of annual percents of enrolments per district was estimated for the period between 2008 and 2010 for possible comparison. Annual percent of enrolments is the total number of women who paid the fixed premium during pregnancy divided by the expected number of pregnancies in the district. It is possible to have >100% for a district, which receives women from other district.

c Three categories of % were generated: < 50%, 50-80% and >80%.

Table 3 shows the characteristics of pregnant women by non-intervention and intervention groups before and after the implementation of the ORI. We observed that before and after introduction of the ORI, women of the intervention group (district with ORI) were likely to live in Nouakchott, in the western part of the country, or in an urban setting. It also arose from Table 3 that those women had better access to high level health facilities (hospital), and were slightly better educated and less poor.

**Table 3:** Characteristics of pregnant women by non-intervention and intervention groups before and after the implementation of the obstetric risk insurance

	Non intervention group (no ORI)				Intervention group (ORI)			
	Before		After		Before		After	
	*N = *3774		*N = *2271		*N = *2551		*N = *1818	
	*N* (%)		*N* (%)		*N* (%)		*N* (%)	
**Health district of residence**								
Nouakchott	364	(9.6)	176	(7.7)	564	(22.1)	603	(33.2)
Zone 1 (West)	635	(16.8)	307	(13.5)	1088	(42.6)	829	(45.6)
Zone 2 (South)	1987	(52.6)	1206	(53.1)	1274	(49.9)	644	(35.4)
Zone 3 (North)	1150	(30.5)	758	(33.4)	189	(7.4)	345	(19.0)
Missing data	2	(0.1)	.		.		.	
Highest level of Health facilities in the health district of residence								
Post	42	(1.1)	28	(1.2)	0	(0.0)	0	(0.0)
Centre	2201	(58.3)	1282	(56.5)	1565	(61.3)	976	(53.7)
Hospital	1529	(40.5)	961	(42.3)	986	(38.7)	842	(46.3)
Missing data	2	(0.1)	0	.	.		.	
Highest level of Health facilities in the municipality of residence								
Post	1974	(52.3)	1282	(56.5)	964	(37.8)	694	(38.2)
Centre	725	(19.2)	450	(19.8)	745	(29.2)	540	(29.7)
Hospital	985	(26.1)	518	(22.8)	839	(32.9)	545	(30.0)
Missing data	90	(2.4)	21	(0.9%)	3	(0.1)	39	(2.1)
Rural versus urban settings								
Rural	2389	(63.3)	1620	(71.3)	1093	(42.8)	742	(40.8)
Urban	1384	(36.7)	650	(28.6)	1458	(57.2)	1076	(59.2)
Missing data	1	(0.0)	1	(0.0)	.		.	
Wealth quintiles of households								
Q1	1884	(49.9)	960	(42.3)	1048	(41.1)	586	(32.2)
Q2	809	(21.4)	554	(24.4)	509	(20.0)	311	(17.1)
Q3	322	(8.5)	160	(7.0)	284	(11.1)	171	(9.4)
Q4	90	(2.4)	100	(4.4)	135	(5.3)	163	(9.0)
Q5	625	(16.6)	486	(21.4)	544	(21.3)	585	(32.2)
Missing data	44	(1.2)	11	(0.5)	31	(1.2)	2	(0.1)
Maternal age								
Mean age (S.D.)	29	(±9)	29	(±10)	29	(±9)	28	(±9)
Age categories								
<18 yr	123	(3.3)	92	(4.1)	93	(3.6)	80	(4.4)
18–34 year	2800	(74.2)	1623	(71.5)	1856	(72.8)	1362	(74.9)
35 year and more	851	(22.5)	556	(24.5)	602	(23.6)	376	(20.7)
Missing data	–		–		–		–	
Mean parity (S.D.)	4	(±3)	4	(±3)	4	(±3)	4	(±3)
Highest education level								
None	1194	(31.6)	553	(24.4)	726	(28.5)	427	(23.5)
Koranic	975	(25.8)	542	(23.9)	680	(26.7)	363	(20.0)
Primary	1120	(29.7)	802	(35.3)	797	(31.2)	689	(37.9)
Secondary +	475	(12.6)	374	(16.5)	345	(13.5)	337	(18.5)
Missing data	10	(0.3)	–		3	(0.1)	2	(0.1)
**Marital status**								
Married	3438	(91.1)	2094	(92.2)	2295	(90.0)	1648	(90.6)
Not married	336	(8.9)	177	(7.8)	256		170	(9.4)
Missing data	–		–		–		–	
**Multiple pregnancy**	26	(0.7)	37	(1.6)	30	(1.2)	30	(1.7)
Missing data	2089	(55.4)	–		923	(36.2)	527	(29.0)

Foot notes: * Women who delivered a live-born child in the last two years before interview (*n* = 10.261).

Abbreviations: ORI: obstetrical Risk insurance.

As shown in Table 4, there was no global effect of the availability of the ORI on facility-based delivery rates. While rates increased in both non-intervention and intervention groups, no significant difference was observed between them after adjusting on covariates. However, the number of deliveries in health care centres increased more rapidly in districts with ORI than in those with no ORI (adjusted OR = 2.34; 95% CI =1.64–2.32; *P* < 0.05). In contrast, deliveries in referral hospitals increased more rapidly in districts with no ORI than in those with ORI (adjusted OR = 0.33; 95% CI =0.23–0.46; *P* < 0.05) where deliveries decreased. When comparing between household wealth quintiles, the effects were not significant. However, facility-based delivery rates significantly increased more rapidly for educated women living in districts with no ORI as compared with those living in districts with ORI (OR = 0.54; 95% CI = 0.33–0.89; *P =* 0.038).

**Table 4:** Rates of facility-based delivery.

	Non interventiongroup (no ORI)			Intervention group (ORI)			Effect of the availability of the ORI		
	Before	After	Diff.	Before	After	Diff.	Adjusted absolute risk difference(95% CI) ^a^		Adjusted Odds Ratios (95% CI) ^b^
	*number (%)*		*%*	*number (%)*		*%*			
All pregnant women									
Total No.	2968	2250		2031	1758				
Facility-based delivery. No. (%)	1389 (46.8)	1267 (56.3)	9.5	1233 (60.7)	1286 (73.2)	12.4	−0.04 [-0.09; 0.13]	0.86 [0.63; 1.21]	
in health post	187 (6.3)	147 (6.5)	0.2	144 (7.1)	126 (7.2)	0.1	0.06 [0.02; 0.09]	1.61 (0.90-2.91)	
in health center	451 (15.2)	452 (20.1)	4.9	490 (24.1)	510 (29.0)	4.9	0.14 (0.06; 0.22)	2.34 (1.64-2.32)*	
in referral hospital	650 (21.9)	625 (27.8)	5.9	541 (26.6)	600 (34.1)	7.5	−0.22 (-0.32;-0.11)	0.33 (0.23; 0.46)*	
Women by level of education									
Non educated									
Total No.	1696	1083		1120	766				
Facility-based delivery. No. (%)	572 (33.7)	465 (42.9)	9.2	510 (45.5)	475 (62.0)	16.5	−0.10 [-0.03; -0.07]	1.18 [0.79; 01.80]	
Educated									
Total No.	1262	1167		908	990				
Facility-based delivery. No. (%)	812 (64.3)	802 (68.7)	4.4	721 (79.4)	810 (81.8)	2.4	0.03 [-0.23;-0.85]	0.54 [0.33; 0.89]*	
Women by wealth quintiles of households									
Q1									
Total No.	1476	955		857	585				
Facility-based delivery. No. (%)	388 (26.3)	316 (33.1)	6.8	273 (31.9)	259 (44.3)	12.4	0.10 [0.05; 0.16]*	1.41 [0.88; 2.23]	
Q5									
Total No.	246	237		211	248				
Facility-based delivery. No. (%)	213 (86.5)	204 (86.2)	−0.3	194 (92.1)	231 (93.2)	1.1	−0.10 [-0.17; -0.03]*	0.52 [0.21; 1.30]	

Abbreviations: ORI: Obstetric risk insurance; No: number; Diff: difference.

a The adjusted absolute risk difference represents adjusted differences between group-specific changes over time and was estimated with the use of generalized linear model.

b The adjusted odds ratios for the interaction between groups (intervention vs. non intervention) and time (before vs after the implementation of the ORI) were estimated with the use of the multivariate generalized estimating-equations extension of logistic regression models.

* *P* < 0.05 were considered to indicate statistical significance.

Subgroup-specific effects were reported when a significant interaction with a subgroup variable was detected. p value of the three-way interaction for level of education*time*intervention was 0.034.

Furthermore, the availability of the ORI had no positive significant effect on antenatal and postnatal care attendance after adjusting on covariates (Table 5). Only qualified antenatal care increased with ORI (OR = 1.53; 95% CI = 1.12–2.06; *P =* 0.008). In contrast, rates of caesarean delivery and modern contraceptives significantly increased more rapidly in districts with no ORI (for caesarean delivery: OR = 0.42; 95% CI = 0.22–0.78; *P =* 0.006 and for modern contraceptive use: OR = 0.42; 95% CI = 0.27–0.68; *P* < 0.001). The effects on secondary outcomes related to maternal health service use were significantly different between educated and non-educated women or related to the household wealth. There was a significant increase in qualified ANC for wealthiest women and a diminution of early neonatal death in the poorest and non-educated women having no access to ORI (see Appendix 1). The increase of caesarean section was lower after ORI implementation in wealthy women.

**Table 5:** Rates of health services utilization and neonatal mortality.

	Non intervention group			Intervention group			Effect of the availability of the ORI	
	Before	After	Diff.	Before	After	Diff.	Adjusted absolute risk difference (95% CI) ^a^	Adjusted Odds Ratios (95% CI)^b^
	*Number (%)*			*Number (%)*				
Antenatal care attendance								
One antenatal visita at least (ANC1)								
Total no.	3684	2250		2547	1779			
ANC1. no. (%)	2782 (75.5)	1867 (82.9)	7.5	1967 (77.2)	1520 (85.4)	8.2	−0.05 [-011; 0.01]	0.94 [0.68; 1.31]
Four antenatal visits at least (ANC4)								
Total no.	908	2250		1110	1270			
ANC4. no. (%)	134 (14.8)	1145 (50.9)	36.1	225 (20.3)	712 (56.1)	35.8	0.00 [-0.05; 0.05]	0.78 [0.58; 1.05]
Antenatal visit with qualified staff (ANQ)								
Total no.	3684	2250		2548	1779			
ANQ. no. (%)	2293 (62.4)	1828 (81.2)	19.2	1412 (55.4)	1500 (84.3)	28.8	0.06 [0.01; 0.11]	1.53 [1.12; 2.10]*
Caesarean delivery (CD)								
Total no.	3684	2250		2546	1779			
CD. no. (%)	18 (0.49)	184 (8.2)	7.7	50 (1.9)	114 (6.4)	4.4	−0.02 [-0.04; 0.01]	0.42 [0.22;0.78]*
Postnatal care attendance (PNC)								
Total no.	2924	2250		2020	1746			
PNC. no. (%)	334 (11.4)	755 (33.6)	22.1	325 (16.1)	539 (30.9)	14.8	−0.01 [-0.07; 0.05]	1.00 [0.73; 1.36]
Family planning (FP). use of modern contraceptives								
Total no.	11348	7799		7009	6875			
FP. no. (%)	389 (3.4)	417 (5.4)	1.9	355 (5.1)	444 (6.5)	1.4	−0.04 [-0.06; 0.02]	0.42 [0.26; 0.68]*
Neonatal mortality								
Early death up to 7 days								
Total no.	1624	2250		1626	1291			
Early deaths. no. (%)	49 (3.0)	38 (1.7)	−1.3	35 (2.2)	20 (1.6)	−0.6	0.01 [0.00; 0.02]	1.67 [0.74; 3.80]
Late death up to 28 days								
Total no.	1624	2250		1626	1291			
Late deaths. no. (%)	60 (3.7)	43 (1.9)	−1.8	40 (2.5)	26 (2.0)	−0.45	0.02 [0.01; 0.03]	2.13 [1.00; 4.54]*

Abbreviations: ORI: Obsteric risk insurance, Diff: difference.

a The adjusted absolute risk difference represents adjusted differences between group-specific changes over time and was with the use of generalized linear model.

b The adjusted odds ratios for the interaction between groups (intervention vs. non intervention) and time (before vs after the implementation of the ORI) were estimated with the use of the multivariate generalized estimating-equations extension of logistic regression models.

* P values of less than 0.05 were considered to indicate statistical significance.

As shown in Table 5, availability of the ORI scheme in the district of residence did not decrease early or late neonatal mortality rates. On the contrary, late neonatal mortality rates globally decreased more rapidly in districts with no ORI as compared with districts with ORI (OR = 2.13; 95% CI = 1.00–4.54; *P =* 0.049).

## Discussion

Our findings arising from the merging of household health surveys in Mauritania prevent us to demonstrate a causal link between the availability of the ORI and the increase in maternal healthcare services utilization or the decrease in neonatal mortality observed in the study time period. This was found in Renaudin et al in 2007 and 2008 and in Vinard 2011 studies, but these authors failed to control for concurrent augmentation on health service utilization, reflecting secular trends in the country. Although most indicators of health care use were not significantly modified by the ORI intervention, we may attribute the increase in birth deliveries of proximity in local health centre versus its decrease in regional hospitals to the availability of the ORI. This could be explained by the fact that women may prefer to deliver where they received their ANC and other examinations offered by the ORI (laboratory and echography) because they are more familiar with the health facility and the health workers. Surprisingly, caesarean delivery rates and use of modern contraceptives increased more rapidly in districts where the ORI was not available compared with those with ORI where standards of medical practice may be more controlled.

Until now, no quantitative study has been carried out to evaluate with rigorous methods the long-term impact of the ORI at the community level. Vinard (2011) observed an increase in deliveries in health care facilities in one moughataa. Data from intermittent community surveys (ICSs), here from DHS, EMIP and NSIMM, were useful to assess a global impact of the availability of the ORI scheme as well as a specific impact in some vulnerable strata of the population. The representativeness of these data and the large sample of women in both groups and study periods is one strength of our study. Indeed, the sample size was also big enough for comparative analyses to evaluate a shift from 50 to 80% of facility-based deliveries.

That ‘non causal’ relationship is probably linked to concurrent co-interventions that were carried out nationwide between 2000 and 2011 and may hide the own effect of the ORI package that has covered so far a limited number of health facilities. Indeed, during this time period a series of national and international actions took place to reinforce the quantity and quality of human resources, broaden geographical distribution of medical staff and health facilities, lead construction or rehabilitation and equipment of structures, and contribute to enhance availability and access of obstetric care in health facilities. Appendix 2 presents a list of some concurrent interventions that could have interfered with the impact of the ORI. For example, from 2004 to 2007 the ACCESS/West Africa program aimed at supporting best practices in maternal and newborn health in Mauritania. This program was able to train doctors to strengthen capacity in obstetric surgery, including caesarean sections. The ACCESS program also supported the revision and pre-testing of new norms, policies and training materials in emergency obstetric and newborn care. In 2008 UNFPA, the Spanish Agency for International Cooperation, WHO and UNICEF initiated a policy to face the lack of obstetric and gynaecology specialists in regions outside of Nouakchott. Some doctors were sent abroad for training and some foreign doctors were, recruited to practice in Mauritania. A National Health Service map has been then introduced, reinforcing of national, regional and peripheral health facilities for procurement and managements of drugs at all levels of the health pyramid.

Some socio-demographic changes in Mauritania after 2002 may also explain the overall rise in maternal health service utilization. The shift from a nomadic to a semi-nomadic or sedentary lifestyle coupled with a rural to urban migration in a great part of the Mauritanian population have probably resulted into a change in health-seeking behaviour and an increase in health-care service utilization nationwide (Okeibunor *et al.* 2013; Lindstrom and Muñoz-Franco 2006).

The dramatic increase we observed in caesarean section rates (from 0.49 to 8.18%) in the districts with no ORI between 2002 and 2010 is quite surprising. Even though the co-interventions cited above could partly explain that increase by greater training and/or decentralization of doctors trained in surgery nationwide during that time period. The fact remains that this increase was very important (+ 8%) and limited to districts with no ORI scheme. Although we do not have qualitative data to support our hypotheses yet, we suggest that in districts where the ORI was available, that the scheme may have contributed to rationalize the indication for a caesarean delivery. Indeed the health facilities offering the ORI have the obligation to monitor and report on a regular basis any information concerning women’s pregnancy, birth delivery and obstetrical practices. Moreover, the ORI entails management, evaluation and surveillance of the medical staff. The fact that any activity must be cautiously reported and caesarean interventions are covered by the insurance when medically necessary only, it may prevent from the overuse of caesarean interventions in low-risk women. Although a rate of 8.18% in districts without ORI remains in the safe and optimal range 10% suggested by WHO, caesarean delivery rates dramatically increased among women living in Nouakchott (from 0.5% to 26.1%) or in household with the richest quintile (0.8–17.9%). This spectacular trend in the districts with no ORI let us to suggest that unnecessary caesarean deliveries could occur without regular monitoring of practices.

Our study has got several limitations however. Firstly, we were not able to know whether the respondent women subscribed to the ORI scheme. Accessible information was limited to the district level, i.e. the date the ORI was implemented and annual adhesion rate among the expected pregnant women. We assumed that a woman who lived in a district where the ORI was available had a better access to join the ORI scheme. However, the level of adhesion reached 80% and more in only eight out of 15 participating districts. To remediate to that different level of women exposure to the ORI, we adjusted our models on the mean percent of adhesion per district of residence. Furthermore, there could be a contamination of the non-intervention group, which could lead us to underestimate the real effect of the ORI. Indeed, the women living in a district without ORI may have joined but in the neighbouring district. Secondly, it remains possible that we have underestimated the use of maternal health services. Indeed, the only pregnancies with newborns alive were taken into account from ICSs and maybe the use of health care services was different among women with newborns alive as compared to those with a stillbirth. We could also question whether women with access to the ORI used more maternity services than other women, resulting in higher maternal survival bias. Finally, although our analyses were adjusted on covariates, districts without or with ORI may be different in unmeasured and systematic ways. For example, the districts participating to the ORI scheme may have been favoured compared to the others. In this case, it is very likely that use of health care services was higher than elsewhere. To control for this bias, the use of difference-in-difference approaches adjusted with covariates, were applied in comparison models.

Global evidence points out seeking or utilization of health care services are shaped by social, cultural and economic environment. Indeed, seeking and utilization of health care services is a phenomenon, which combines a complex network of determinants, including availability and access to health care services, but also the interplay between individual characteristics, community health beliefs and socio-cultural norms and the entire health care system. Context shapes health care choices and thus directly influences health care utilization. Inadequate financial resources and an under-resourced health care system in Mauritania contribute to delay in accessing medical facilities. Using qualitative study will fill a gap in understanding the perception of the ORI scheme and the reasons for the enrolment among pregnant women in Mauritania (De Allegri and Sauerborn 2007; Ridde *et al.* 2014). Further research is needed to assess the impact of the ORI scheme in terms of reduction of out-of-pocket expenditure and quality of care.

## Conclusion

In general, the availability of the ORI scheme did not cause the expected increase in facility-based deliveries. No significant association between the implementation of the ORI scheme and increased utilization of maternal health services was detected, except for delivery in local health care centres and qualified ANCs. In addition, the ORI scheme was not found to have a significant positive effect on neonatal mortality. The availability of the ORI has not fully met its objective of attracting all pregnant women towards better access to facility-based health care and it seems that some strata of women benefitted more than others. To better understand and complement our findings, a qualitative study that will explore the context and complexity of intervention implementation and how socio-cultural dynamics influence health-related behaviours, including the decision to enrol in the ORI scheme, will be of great importance.

## Appendix 1. Changes in maternal health services utilization and neonatal mortality in non intervention and intervention groups after implementation of the obstetrical risk insurance and by stratifying by women’s education and wealth quintiles of household

**Table:**

	Non intervention group			Intervention group				
	Before N (%)	After N (%)	Δ	Before N (%)	After N (%)	Δ	DID	OR
**ANC**								p = 0.090*^m^
Non educated	1454 (68.4)	859 (79.3)	11.0	978 (69.7)	619 (79.9)	10.2	DID: -0.07 [-0.15; 0.00]	OR: 0.80 [0.53; 1.21]
Educated	1321 (85.4)	1008 (86.4)	1.0	988 (86.6)	900 (89.8)	3.2	DID: 0.02 [-0.03; 0.06]	OR: 1.40 [0.79; 2.47]
								p = 0.664
Q1	1178 (63.8)	718 (76.0)	12.2	640 (61.3)	417 (71.2)	9.9	DID: -0.06 [-0.14; 0.03]	OR: 0.67 [0.43; 1.08]
Q5	285 (93.2)	218 (92.2)	−1.0	249 (92.1)	238 (94.9)	2.8	DID: 0.00 [-0.05; 0.05]	OR: 1.42 [0.49; 4.15]
**ANC_qualified staff**								p = 0.272
Non educated	1130 (77.7)	831 (96.7)	19.0	655 (66.9)	610 (98.6)	31.6	DID: 0.04 [-0.03; 0.10]	OR: 0.80 [0.53; 1.21]
Educated	1156 (87.5)	997 (98.9)	11.4	756 (76.5)	889 (98.8)	22.26	DID: 0.10 [0.05; 0.15]	OR: 1.90 [1.18; 3.06]**
								p = 0.000**
Q1	924 (78.4)	698 (97.2)	18.8	441 (68.9)	412 (98.8)	30.0	DID: -0.02 [-0.09; 0.05]	OR: 0.69 [0.43; 1.13]
Q5	258 (89.8)	217 (98.7)	8.8	193 (76.9)	238 (99.1)	22.2	DID: -0.24 [0.18; 0.30]**	OR: 3.67 [1.68; 7.94]**
**ANC 4+**								p = 0.720
Non educated	62 (25.3)	502 (63.8)	38.5	91 (33.1)	270 (66.1)	33.0	DID: 0.04 [-0.03; 0.10]	OR: 0.88 [0.57; 1.36]
Educated	72 (21.3)	643 (58.4)	37.1	134 (22.5)	442 (57.5)	34.93	DID: -0.02 [-0.04; 0.04]	OR: 0.70 [0.46; 1.07]
								p = 0.218
Q1	34 (19.5)	407 (56.7)	37.2	43 (18.4)	200 (59.5)	41.1	DID: 0.05 [0.01; 0.11]	OR: 1.04 [0.57; 1.87]
Q5	19 (27.7)	142 (67.0)	39.2	36 (41.0)	129 (66.1)	25.11	DID: -0.12[-0.62; 0.07]*	OR: 0.52 [0.28; 0.95]*
**Caesarean section**								p = 0.421
Non educated	7 (0.3)	48 (4.4)	4.1	24 (1.7)	25 (3.2)	1.6	DID: -0.03 [-0.04; 0.01]	OR: 0.27 [0.10. 0.75]*
Educated	11 (0.7)	136 (11.7)	10.9	26 (2.3)	89 (8.9)	6.6	DID: -0.02 [-0.05; 0.01]	OR: 0.53 [0.23; 1.22]
								p = 0.015*
Q1	5 (0.3)	25 (2.7)	2.4	5 (0.5)	14 (2.4)	(1.9)	DID: 0.01 [-0.03; 0.03]	OR: 0.60 [0.50; 13.57]
Q5	3 (0.8)	51 (17.9)	17.1	9 (3.3)	22 (10.3)	(7.0)	DID: -0.10[-0.15;-0.05]*^m^	OR: 0.25 [0.08; 0.79]*
**PNCs**								p = 0.706
Non educated	140 (8.4)	331(30.6)	22.2	130(11.7)	205 (26.9)	15.2	DID: 0.01 [-0.07; 0.05]	OR:1.05 [0.67; 1.66]
Educated	194 (15.6)	424 (36.3)	20.7	194 (21.4)	332 (33.8)	12.3	DID: -0.01 [-0.08; 0.08]	OR: 0.95 [0.62; 1.45}
								p = 0.131
Q1	87 (6.0)	261 (27.6)	21.6	91 (10.7)	156 (26.7)	16.1	DID: 0.07 [0.01; 0.14]	OR: 1.69 [0.93; 3.09]*^m^
Q5	74 (23.7)	108 (40.7)	17.1	57 (23.8)	77 (32.0)	8.2	DID: -0.06 [-0.18; 0.06]	OR: 0.71 [0.39; 1.31]
**Early death (0-6 d)**								p = 0.044**
Non educated	34 (3.4)	14 (1.3)	−2.1	17 (1.9)	8 (1.4)	−0.4	DID: 0.03[0.01; 0.04]*^m^	OR: 3.59 [0.98; 13.2]*
Educated	15 (2.4)	24 (2.1)	−0.3	18 (2.5)	12 (1.7)	−0.9	DID: -0.01[-0.02; 0.01]	OR: 0.70 [0.22; 2.25]
								p = 0.027*
Q1	22 (2.8)	7 (1.7)	−1.1	12 (2.0)	8 (2.0)	0.0	DID: 0.02[0.01; 0.04]	OR: 3.52 [0.85; 14.7]*^m^
Q5	5 (3.0)	4 (3.5)	0.6	5 (1.9)	8 (1.2)	−0.7	DID: -0.02[-0.04; 0.00]	OR: 0.40 [0.06; 2.50]
**Late death (7-28 d)**								p = 0.174
Non educated	38 (3.8)	19 (1.8)	−2.1	21 (2.3)	11 (1.9)	−0.3	DID: 0.03[0.01; 0.04]*	OR: 3.11 [1.00; 9.69]*
Educated	22 (3.5)	19 (2.1)	−1.4	19 (2.7)	15 (2.1)	−0.6	DID: 0.00[-0.02; 0.02]	OR: 1.21 [0.41; 3.56]
								p = 0.06*^m^
Q1	27 (3.4)	9 (1.0)	−2.5	16 (2.5)	9 (2.1)	−0.4	DID: 0.02[0.01; 0.04]	OR: 3.26 [0.89; 12.0]*^m^
Q5	7 (3.7)	4 (3.7)	−0.0	5 (2.2)	1 (1.5)	−0.8	DID: -0.01[-0.01; 0.01]	OR: 0.63 [0.11; 3.51]

Abbreviations: DID, difference-in-differences; OR, odd ratio.

a two-way interaction: time *intervention.

b *P* value of the three-way interaction: subgroup*time*intervention.

* ≤ 0.05; ** ≤ 0.01; *** *P* ≤0.001.

*m marginal level of significance (0.05 < m < 0.10)

## Appendix 2. List of available concurrent strategies/interventions that took place in the country during the study time period and their hypothetical effects on increased use of maternal and perinatal services and decreased neonatal mortality

**Table:**

Date of implementation	Strategy/intervention	Main goal(s)	Possible effect on use of maternal and perinatal services	Possible effect on Neonatal mortality
2002	Poverty reduction strategy	To increase of medical staff	x	X
		Obstetrician/gynaecologist		
2003	Make pregnancy safer program	To improve equipment for hospitals in Nouakchott and Nouadhibou	x	X
2003	Ministry of Health strategy	To start incentive scheme of bonus for working i n regions outside of Nouakchott	x	X
2004	ACCESS/West Africa program	To improve best practices in maternal and newborn health with training of medical staff		X
2005-2015	National Health and Social Development Policy originated from:the third poverty reduction strategy framework (CSLP3) and the health-related Millennium Development Goals in Mauritania’s health sector (WHO 2004)	To enhance access and performance of the heath system: to rehabilitate or construct new health structures to implement a new strategic human resources to improve health and nutritional status	x	X
2008	Ministry of Health strategy	Guidelines for use of maternal health services	x	X
2008	Common initiative from The United Nation Population Fund (UNFPA), the Spanish Agency for International Cooperation (AECID), WHO and UNICEF	Reinforcement medical staff	x	X
2009	Ministry of Health	Opening of a new Public Health school	x	X
